# Is Early Enteral Nutrition Initiated Within 24 Hours Better for the Postoperative Course in Esophageal Cancer Surgery?

**DOI:** 10.4021/jocmr1665w

**Published:** 2013-12-13

**Authors:** Naoko Manba, Yu Koyama, Shin-ichi Kosugi, Takashi Ishikawa, Hiroshi Ichikawa, Masahiro Minagawa, Takashi Kobayashi, Toshifumi Wakai

**Affiliations:** a Division of Digestive and General Surgery, Niigata University Graduate School of Medical and Dental Sciences, Niigata, Japan

**Keywords:** Early enteral nutrition, Esophageal cancer, Infectious complication, Pneumonia

## Abstract

**Background:**

Early enteral nutrition within 24 h after surgery has become a recommended procedure. In the present study, we retrospectively examined whether initiating EN within 24 h after esophagectomy improves the postoperative course.

**Methods:**

Among 103 patients who underwent thoracic esophagectomy for esophageal cancer, we enrolled the cases in which EN was initiated within 72 h after surgery. The patients were divided into two groups: EN started within 24 h (Group D1) and EN started at 24 - 72 h (Group D2-3). Clinical factors including days for first fecal passage, dose of postoperative albumin infusion, difference in serum albumin between pre- and postoperation, incidence of postoperative infection, and use of total parenteral nutrition were compared. Statistical analyses were performed by the Mann-Whitney U test and Chi square test, with significance defined as P < 0.05.

**Results:**

There was no significant difference between the groups in clinical factors. While pneumonia was significantly more frequent in Group D1 than in Group D2-3 (P = 0.0308), the frequency of infectious complications was comparable between the groups.

**Conclusion:**

Initiating EN within 24 h showed no advantage for the postoperative course in esophageal cancer, and thus EN should be scheduled within 24 - 72 h, based on the patient condition.

## Introduction

Several studies have advocated early enteral nutrition (EN) in various patient groups including critically ill patients [1-3], acutely hospitalized medical patients [4, 5], burns patients [6-9], trauma patients [10, 11], and septic patients [12, 13]. Moreover, patients undergoing major surgery have gained proven advantage from EN started at an earlier stage after surgery [14-20]. These studies have demonstrated several advantages of early EN initiated postoperatively as follows: lower incidence of septic complications [14], shorter hospital stay, and decreased weight loss [18]. Classically, the term “early” was defined as EN started within 3 days after admission or surgery [9]; however, “early” is more recently considered as EN started within 24 - 48 h after admission or surgery [1]. Further, EN started within 24 h after surgery or trauma has been shown some benefits such as reduction in mortality, septic complication, and life-threatening complications [11, 21-23]. Transthoracic esophagectomy with 3-field lymphadenectomy (TTE-3FL) for esophageal cancer is one of the most invasive procedures among gastrointestinal surgeries, and patients undergoing TTE-3FL are unable to gain nutrition by mouth within the first few days after surgery. Thus, postoperative EN and/or parenteral nutrition have become routine management in such cases, and recent studies demonstrated that EN initiated within 24 - 48 h after esophagectomy reduced the length of hospital stay [24, 25], postoperative morbidity [20], and the rate of life-threatening complications [22]. Moreover, some clinical guidelines on EN for surgical patient have also recommended early EN initiated within 24 h after gastrointestinal surgery, including esophagectomy [26]. However, almost all of these studies described the superiority of EN initiated within 24 h in comparison with total parenteral nutrition (TPN), thus it remains unclear whether EN initiated within 24 h is more beneficial in comparison with EN initiated 24 - 72 h after trauma or surgery, including esophagectomy. Moreover, because some studies could not show any clinical benefits with routine postoperative EN after esophagectomy [27-29], the validity of early EN after esophagectomy, especially within 24 h, is still controversial [30].

The aim of the present retrospective study was to examine whether early EN initiated within 24 h after esophagectomy is superior to EN initiated 24 - 72 h in terms of the postoperative course.

## Materials and Methods

### Patient selection

Patients who underwent TTE-3FL for esophageal cancer at Niigata University Medical and Dental Hospital during 1996 - 2010 (103 patients in total) were entered into the present retrospective chart review. Among these cases, we analyzed the data from 42 patients for whom EN was initiated within 3 postoperative days. The study protocol was approval by the Institutional Review Board for Clinical Research. The patients were divided into two groups based on the start date of EN administration as follows: Group D1 comprised patients started on EN within 24 h after surgery, and Group D2-3 comprised patients started on EN within 24 - 72 h after TTE-3FL. The EN was started with an initial dose of 200 - 250 mL of oligomeric or polymeric formula (1 kcal/mL) under 10 - 20 mL/h from the jejunostomy. In both groups, the EN dose was gradually increased every 12 - 24 hours if there were no problems related to the EN, to reach a maximum dose at day 5 - 6 after starting EN. Peripheral intravenous infusions of 4.3% glucose with electrolyte solutions were also supplied in both groups. TPN was introduced if the EN could not be started by postoperative day 5.

### Clinicopathological assessment

Clinicopathological factors were compared between the groups as follows: age, sex, tumor stage according to the tumor-node metastasis classification of the International Union Against Cancer (6th edition), days for first fecal passage, the dose of postoperative albumin infusion used, difference in serum albumin values between day 7 and the presurgical assessment (Δalb), duration of systematic inflammatory response syndrome (SIRS), incidence of postoperative complications, and use of TPN. SIRS was diagnosed according to the criteria of the ACCP-SCCM Consensus Conference Committee [28]. For the diagnosis, at least two of the following criteria had to be fulfilled: systolic blood pressure < 90 mmHg, tachycardia > 90/minutes, respiratory rate > 20/minutes or peripheral arterial CO_2_ tension (PaCO_2_) < 32 mmHg, temperature > 38.0 °C or < 36.0 °C, leukocytosis > 12,000/µL or leukopenia < 4,000/µL or 10% immature (band) forms.

Postoperative complications were also retrospectively reviewed from patient records, and complications were classified as either non-infectious or infectious. In this study, a mechanical complication was defined as that directly due to a failure in the surgical procedure, and an infectious complication was one accompanying infection. Recurrent nerve palsy was assessed clinically and endoscopically, and subclinical recurrent nerve palsy was also judged as positive.

### Statistical analysis

The statistical analyses were performed using the Mann-Whitney U test and Chi square test. Statistical significance was defined as P < 0.05.

## Results

### Pre- and peri-operative clinicopathological features

Among the 42 patients who received early EN, 15 patients were categorized into Group D1, and 27 patients were categorized into Group D2-3 (Table 1). There was no significant difference in mean age, sex, preoperative nutritional conditions expressed by body mass index (BMI), body weight, or serum albumin values between Group D1 and Group D2-3. Tumor stage was also comparable between the groups; however, preoperative chemotherapy was significantly more frequent in Group D1 patients than in Group D2-3 (P = 0.0129). Postoperative TPN use was comparable between the groups.

**Table 1:** Pre- and Peri-Operative Clinicopathological Feature

	Total	D1 (n = 15)	D2-3 (n = 27)	P value (Mann-Whitney test)
Age (years)	61.5 ± 6.6	60.5 ± 6.3	62.1 ± 6.8	0.5373
BMI (kg/m^2^)	21.8 ± 3.1	20.9 ± 2.4	22.4 ± 3.4	0.1246
Body weight (kg)	57.7 ± 9.8	55.1 ± 9.5	59.1 ± 9.9	0.1488
Albumin (mg/dL)	4.0 ± 0.4	3.9 ± 0.5	4.0 ± 0.3	0.6600
Operative time (min)	470 ± 83	475 ± 83	467 ± 85	0.9060
Blood loss (mL)	676 ± 280	679 ± 273	673 ± 290	0.7528

				**P value (Chi square test)**

Sex				0.8950
Male	36	13	23	
Female	6	2	4	
Stage				0.1536
0	2	1	1	
I	15	4	11	
IIA	7	1	6	
IIB	2	1	1	
III	8	6	2	
IVA	3	0	3	
IVB	5	2	3	
Preop. chemotherapy				0.0129
No	22	4	18	
Yes	20	11	9	
Postop. TPN use				0.2348
No	32	13	19	
Yes	10	2	8	

TPN: total parenteral nutrition.

### Postoperative complications and mortality

Postoperative complications were observed in 36 patients (85.7%), but there was no significant difference in morbidity between Group D1 and Group D2-3. Further analysis was performed by dividing the complications into non-infectious and infectious. The groups showed no difference in non-infectious complications. Frequency of postoperative infectious complications were also comparable between the groups; however, pneumonia was significantly more frequent in Group D1 compared with Group D2-3 (P = 0.0308). The mortality rate among all patients was comparable (Table 2).

**Table 2:** Postoperative Complications and Mortality

	Total	D1 (n = 15)	D2-3 (n = 27)	P value
Postop. complications	36	13	23	0.8954
Non-infectious	21	8	13	0.7474
Recurrent nerve palsy	22	8	14	0.9266
Anastomotic dehiscence	14	5	9	0.9999
Tracheal damage	1	1	0	0.6657
Aortic rupture	1	1	0	0.6657
Infectious	17	6	11	0.9626
Pneumonia	7	5	2	0.0308
Wound infection	7	3	4	0.6657
Sepsis	3	0	3	0.4749
Mortality	1	1	0	0.6657

### Postoperative outcomes between Group D1 and D2-3

There was no significant difference in postoperative EN calories received (kcal/kg) between the groups. First fecal passage, postoperative albumin infusion, Δalb, duration of SIRS and respirator use, and the length of postoperative hospital stay were also comparable between Group D1 and Group D2-3 (Table 3).

**Table 3:** Postoperative Outcomes of Early EN Initiated Within 24 h and During 24 - 72 h

	Total	D1 (n = 15)	D2-3 (n =27)	P value
EN calorie (kcal/kg)	28.4 ± 8.5	26.5 ± 8.1	29.4 ± 8.6	0.3249
First fecal passage (day)	6.5 ± 2.3	4.8 ± 1.7	5.5 ± 1.6	0.2535
Albumin infusion (mL)	83.1 ± 61.7	79.3 ± 66.9	85.0 ± 59.8	0.8031
Δalbumin (mg/dL)	-1.18 ± 0.5	-1.3 ± 0.6	-1.1 ± 0.4	0.4833
SIRS duration (day)	4.0 ± 5.1	5.8 ± 8.3	3.0 ± 2.0	0.9525
Respirator duration (day)	3.7 ± 7.3	5.9 ± 11.9	2.6 ± 1.6	0.5636
LOH (day)	54.2 ± 52.0	68.9 ± 74.0	46.0 ± 33.4	0.6087

Δalbumin: difference in serum albumin values between postoperative day 7 and pre-operation; SIRS: systematic inflammatory response syndrome; LOH: length of hospital stay.

## Discussion

In general, esophageal cancer patients are frequently malnourished due to several possible factors including esophageal stenosis, their habits, preoperative systemic chemotherapy, or the systemic effect of their neoplasm [31]. However, preoperative nutritional status was not poor in our study, based on nutritional parameters indicated by BMI, body weight, and serum albumin concentration, showing a good-nutrition preference. In addition, the preoperative nutritional conditions were comparable between groups in this study. Surgical stress in both groups also seemed to be equivalent because there was no significant difference in operative time or blood loss between Group D1 and Group D2-3.

Unlike some previous studies [22, 27], our findings showed no advantage of starting EN within 24 h after esophagectomy for reducing postoperative morbidity or life-threatening complications. However, there was some evidence in the present study of reduced complications with the early EN initiated within 24 h. The discrepancy in morbidity results between past studies and ours might be due to the different nutritional support methods used, in that most of the previous studies compared early EN versus TPN, which might have resulted in the superior results with EN. However, our study compared early EN initiated within 24 h versus EN initiated during 24 - 72 h, with no apparent differences evident in postoperative morbidity or life-threatening complications between the groups.

In addition, while there was no significant difference in infectious complications among our patient cohort, Group D1 patients showed a significantly higher incidence of pneumonia than those in Group D2-3. EN administered within 24 h after esophagectomy or pancreatoduodenectomy has resulted in impaired respiratory mechanics indicated by significantly lower vital capacity and forced expiratory volume in one second (FEV_1_) compared with controls [20], and indeed, our study did show a high frequency of recurrent nerve palsy in both groups. However, we believe that the strict endoscopic assessment of recurrent nerve palsy in our department might account for this finding, and that most of the cases were in fact subclinical and transient. Moreover, there was no significant association between recurrent nerve palsy and postoperative pneumonia in our study. Therefore, early EN initiated within 24 h might also cause impaired respiratory function such as decreased vital capacity and/or FEV_1_, and thus lead to the high frequency of postoperative pneumonia in Group D1 in our study.

Postoperative outcomes indicated by first fecal passage, albumin infusion, difference in serum albumin between pre-operative and day 7 postoperative measurements were comparable between the two groups in our study, as were supplied EN calories, the duration of SIRS and respiratory management. Therefore, we found no significant benefits specifically associated with early EN initiated within 24 h after esopgagectomy compared with that initiated at 24 - 72 h.

### Conclusion

Administering EN within 24 h showed no further advantage for the postoperative course of patients undergoing surgery for esophageal cancer compared to EN started within 24 - 72 h. We therefore recommend that early EN initiation should be scheduled according to the condition of the patient in the range of 72 h after esophagectomy.
